# Exon Silencing Regulated by a Trio of Short RNA Motifs

**DOI:** 10.1371/journal.pbio.0030173

**Published:** 2005-04-19

Our cells make many more kinds of proteins than can be accounted for by the relatively modest number of genes in our genome. The key to this protein-coding bounty is alternative splicing, in which one or more transcribed exons—nucleotide sequences that code for a specific segment of the protein—are excluded from the final messenger RNA before it is translated into protein. While the majority of human genes are alternatively spliced, little is known about specific RNA sequences that dictate exclusion of these exons. In a new study, Paula Grabowski and colleagues show that three short sequences, two within the excluded exon and one in an adjacent intron, or non-coding nucleotide sequence, trigger exclusion in at least one gene, and probably a large handful of others as well.

Grabowski and colleagues studied this process in a class of proteins essential for brain function called glutamate receptors. As the name implies the glutamate receptors bind to glutamate, the principal excitatory neurotransmitter in the brain. NMDA glutamate receptors, which play a role in memory formation and neuronal development, are composed of multiple subunits. Within the NR1 subunit, exclusion or inclusion of the CI cassette exon has dramatic functional consequences. The CI exon appears in the forebrain but is virtually absent in the hindbrain. How this differential splicing is regulated is poorly understood.

The authors noted an atypical but highly conserved GGGG motif in the intron just downstream from the splice site that ends the CI exon. When they introduced point mutations in this motif, the exon was included up to four times as often. The rate of exclusion, or silencing, could be dramatically increased by the addition of another GGGG tetrad farther inside the intron.

Systematic mutation within the exon identified a pair of UAGG motifs that also promoted exon silencing, an effect that could be enhanced even further by introducing a third, artificial, UAGG. The pair of UAGG tetrads appears to work in combination with the GGGG tetrad, since without the former sequences, the latter had little power to silence CI expression. Silencing is mediated by binding of UAGG to the ribonucleoprotein hnRNP A1, which also apparently interacts with the GGGG within the intron.

The authors next did a series of genomic database searches, to identify these motifs in other genes. They reasoned that if the triad was a common means of exon silencing, it should be overrepresented among genes known to undergo alternative splicing. In more than 90,000 exons in human and mouse genomes, they found 16 with the motif pattern, of which three (19%) were known skipped exons. In contrast, among those without the pattern, the proportion of skipped exons was only 5%. They also found that the GGGG motif by itself was overrepresented among skipped exons, indicating it probably plays a significant role in exon exclusion even without its UAGG partners.

These results alone cannot explain why one cell type includes an exon while another excludes it, since the primary transcript in different cell types is the same. Instead, these differences are likely explained by tissue-specific differences in levels of splicing factors or binding proteins. With such small absolute gene numbers, it is clear that the specific trio identified by Grabowski and colleagues is only one of many likely to regulate exon inclusion. In the search for others, this study indicates the value of bioinformatics strategies that employ not only specific sequences, but also spatial configurations.

